# Bioenhanced degradation of toluene by layer-by-layer self-assembled silica-based bio-microcapsules

**DOI:** 10.3389/fmicb.2023.1122966

**Published:** 2023-02-20

**Authors:** Hongyang Lin, Yang Yang, Yongxia Li, Xuedong Feng, Qiuhong Li, Xiaoyin Niu, Yanfei Ma, Aijv Liu

**Affiliations:** ^1^School of Agricultural Engineering and Food Science, Shandong University of Technology, Zibo, China; ^2^Shandong Academy of Environmental Science Co., Ltd., Jinan, China; ^3^School of Resources and Environmental Engineering, Shandong University of Technology, Zibo, China; ^4^School of Materials Science and Engineering, Shandong University of Technology, Zibo, China

**Keywords:** layer-by-layer self-assembled, silica-based, bio-microcapsules, toluene, environmental tolerance

## Abstract

In this study, micron-sized monodisperse SiO_2_ microspheres were used as sacrificial templates, and chitosan/polylactic acid (CTS/PLA) bio-microcapsules were produced using the layer-by-layer (LBL) assembly method. Microcapsules isolate bacteria from their surroundings, forming a separate microenvironment and greatly improving microorganisms’ ability to adapt to adverse environmental conditions. Morphology observation indicated that the pie-shaped bio-microcapsules with a certain thickness could be successfully prepared through LBL assembly method. Surface analysis showed that the LBL bio-microcapsules (LBMs) had large fractions of mesoporous. The biodegradation experiments of toluene and the determination of toluene degrading enzyme activity were also carried out under external adverse environmental conditions (i.e., unsuitable initial concentrations of toluene, pH, temperature, and salinity). The results showed that the removal rate of toluene by LBMs can basically reach more than 90% in 2 days under adverse environmental conditions, which is significantly higher than that of free bacteria. In particular, the removal rate of toluene by LBMs can reach four times that of free bacteria at pH 3, which indicates that LBMs maintain a high level of operational stability for toluene degradation. Flow cytometry analysis showed that LBL microcapsules could effectively reduce the death rate of the bacteria. The results of the enzyme activity assay showed that the enzyme activity was significantly stronger in the LBMs system than in the free bacteria system under the same unfavorable external environmental conditions. In conclusion, the LBMs were more adaptable to the uncertain external environment, which provided a feasible bioremediation strategy for the treatment of organic contaminants in actual groundwater.

## 1. Introduction

Benzene, toluene, ethylbenzene, and xylene (BTEX) are common organic pollutants that leak into the groundwater and have caused serious pollution to groundwater (Fedorov et al., 2021). Among BTEX, toluene is relatively more soluble in water, with solubility of 535 mg/L at 25°C (El-Naas et al., 2014), and the log Kow of toluene is 2.75, indicating that toluene is more easily distributed in the aqueous phase than in the soil (Šoštarić et al., 2016). Long-term exposure to BTEX compounds has adverse effects on human health (such as damaging the central nervous system) and ecosystem functions (such as inhibiting the survival of earthworms; Picone, 2012). Therefore, the removal of BTEX from groundwater, especially toluene, is essential to ensure the safety of water (Asenjo et al., 2011).

Bioremediation has cost and technical advantages over other treatment technologies (e.g., physical or chemical techniques; Elmrini et al., 2004; Firmino et al., 2015; Gul et al., 2015), and it is considered a promising strategy to remove BTEX from the environment. However, the remediation of BTEX contaminated groundwater by free bacteria is usually limited by the external environment (high pollutant concentration, pH, temperature, salinity). In addition, free bacteria are easily washed away by water, which prevents their settlement (Boufadel et al., 2016) and further weakens the effectiveness of microbial treatment.

Microbial immobilization technology has been shown to be effective in improving the fitness of free bacteria (Lin, 2009; Xin et al., 2013; Miri et al., 2021). Commonly used methods include immobilization of bacteria by physical adsorption or polymeric gel beads (Liu et al., 2012; Lin et al., 2013; Wen et al., 2021). However, the application of microbial immobilization technology is limited due to the weak impact resistance of the physical adsorption method and the poor mass transfer performance of the entrapment of bacteria in gel bead (Seo et al., 2001; Jiang et al., 2022). The LBL microcapsules were prepared by depositing oppositely charged polyelectrolytes onto the surface of sacrificial templates by the LBL method, and the constituent layers were bound together by strong electrostatic interactions (Del Mercato et al., 2010; Zhang et al., 2012). The polyelectrolyte hollow microcapsule technology has been become the focus of attention since it was first reported by Möhwald’s group in 1998 (Donath et al., 1998), and the good biocompatibility makes it a great potential for applications in the biomedicine, catalysis, and food industries (Xuan et al., 2017). In recent years, LBL microcapsules have also been developed for the protection of microorganisms. Deng et al. (2017) prepared chitosan/alginate bio-microcapsules by layer-by-layer (LBL) assembly method and tested for pyrene (PYR) biodegradation under harsh environmental conditions. The results indicated that bacteria in microcapsules treatment gained a much higher tolerance to environmental stress. LBL microcapsules could form complete protection for the microorganisms. In addition, LBL microcapsules have a dense pore structure in the capsule wall, facilitating the capture of substrates and the excretion of metabolites by the microorganisms (Jiang et al., 2009). To a certain extent, it overcomes the disadvantages of traditional microbial immobilization methods.

This study aims to expand the advantages of microbial applications by preparing an environmentally friendly material to help microorganisms cope with the harsh external environment. In this work, LBMs were prepared by the sacrificial template method using micron-sized monodisperse silica (SiO_2_) as the sacrificial template and CTS/PLA as the capsule wall material and were used for the first time for toluene abatement. The tolerance of LBMs to some harsh environmental conditions (i.e., unsuitable initial toluene concentration, pH, temperature, and salinity) was investigated. The mechanism of bacterial protection by LBL microcapsules was inferred by monitoring the changes in relevant enzyme activities and changes in the number of live bacteria in both systems.

## 2. Materials and methods

### 2.1. Materials and organism

The micron-sized monodisperse SiO_2_ microspheres were prepared according to the literature methods (Xing, 2015). CTS and PLA were purchased from Aladdin Chemical Reagent Co. (Shanghai, China; A. R. grade, purity ≥98%). Toluene, carbon disulfide, N,N-dimethylformamide, and catechol were bought from Yantai Yuandong Fine Chemical Co., Ltd. (Yantai, China; A. R. grade, purity ≥98%).

The microorganism used in this study was *Bacillus stercoris* EGI312, which was isolated and purified from activated sludge. The activated sludge was taken from the sewage treatment station of Shandong Chambroad Petrochemicals Co., Ltd. in Binzhou, Shandong, China. The bacteria were cultured in fresh LB medium for 2–3 days to an optical density (OD) of 2. After being centrifuged at 5,000 rpm for 5 min, the bacteria were resuspended with 0.9% NaCl for immobilization according to the method by Deng et al. (2017).

### 2.2. Preparation of LBMs

Based on the principle of LBL, LBMs were prepared by immobilizing EGI312 with CTS and PLA as capsule wall materials and SiO_2_ as templates. Firstly, the SiO_2_ and the bacteria suspension were mixed. The CTS was then added under stirring and it was deposited onto the surface of SiO_2_. Subsequently, the PLA was added, which was further deposited onto the surface of the CTS due to electrostatic forces. The above cycle was repeated to form a multilayer structure, and then the SiO_2_ cores were dissolved in dilute hydrofluoric acid (HF, pH = 5).

### 2.3. Characterization of LBMs

The surface Zeta-potentials of SiO_2_ microparticles after the deposition of the CTS/PLA layer were measured with a Dalven Zeta-potential analyzer. Three parallel measurements were conducted for every sample, and the average values were reported. Morphological analyses of the LBMs were performed by a scanning electron microscope (SEM, Germany) with an acceleration voltage (Acc. 5 kV) and a transmission electron microscope (TEM, Tecnai G2F 20, United States). The structural characteristics of LBMs were characterized by Fourier transform infrared spectroscopy (FTIR, Nicolet 5,700, USA) with a wavenumber from 4,000 to 500 cm^−1^ and X-ray diffraction spectroscopy (XRD, Rigaku SmartLab, Rigaku Co., Japan, CuKα source, operating at 40 mA and 40 kV, wave-length 0.15406 nm, 2θ 5–40°, scanning rate 0.1 s/step, resolution 0.01°/step). Electron microscopy was used to observe the distribution of microorganisms in the LBL microcapsules. The N_2_ adsorption–desorption isotherms of the LBMs were measured by a specific surface area and pore size analyzer (BET, asap246, United States) to characterize the specific surface area and pore size. The specific surface areas were calculated following the Brunauer–Emmett–Teller (BET) method, and the pore-size distributions were analyzed by using the desorption branch isotherms obtained using the density functional theory (DFT) model.

### 2.4. Toluene biodegradation experiments

Batch experiments on toluene degradation by free bacteria and LBMs were carried out in 100 ml shake flasks containing 50 ml of inorganic salt medium with toluene as the sole carbon source. The inorganic salt medium consisted of the following: 4 g L^−1^ K_2_HPO_4_•3H_2_O, 4 g L^−1^ NaH_2_PO_4_•2H_2_O, 2 g L^−1^ (NH_4_)_2_SO_4_, 0.2 g L^−1^ MgSO_4_, 0.01 g L^−1^ CaCl_2_, 0.01 g L^−1^ MnSO_4_•H_2_O, 0.01 g L^−1^ FeSO_4_•7H_2_O. The effects of the initial toluene concentration (300, 400, and 500 mg L^−1^), pH (3, 7, and 10), temperature (10°C, 30°C, and 40°C), and salinity (NaCl, w/V, 0, 2, and 5%) on the toluene biodegradation were investigated in the free bacteria and LBMs systems, respectively. Samples of the free bacteria and LBMs systems were ultrasonically dissolved in isovolumetric carbon disulfide for 5 min, respectively, and the organic phase was separated after static stratification. The concentration of toluene was determined using a gas chromatograph (GC, Agilent Technologies 7890B, United States) equipped with a flame ionization detector (FID, Agilent G4556-64000, United States).

### 2.5. Analysis of the survival and death of the bacteria

The Calcein/PI dye (Calcein/PI Cell Viability/Cytotoxicity Assay Kit, Sangon Biotech, Shanghai, China) was used to stain the bacteria to analyze the ratio of live and dead cells of the free and LBL microencapsulated bacteria through flow cytometry (CytoFLEX, Beckman, Shanghai, China), respectively. The experiments were conducted at an initial toluene concentration of 500 mg/L and other conditions were optimal for bacterial growth (pH = 7, temperature = 30°C， salinity = 0%). The samples were taken during the degradation of toluene (0, 2, 4 days) and the bacteria were collected by centrifugation. After being washed 2–3 times with PBS, the bacteria were mixed with Calcein/PI dye solution (0.2 μl in a proportion of 1:1) and incubated for 15 min, avoiding light at 37°C. Then, the bacteria solution carried out flow cytometry detection. The detection excitation light was 490 nm, and more than 10,000 cells were counted.

### 2.6. Determination of enzyme activity

#### 2.6.1. Preparation of crude enzyme solution

Samples of free bacteria and LBMs systems were taken separately and centrifuged at high speed for 10 min at a low temperature, and then the bacteria were rinsed twice with phosphate buffer (pH 7.0). The cells were crushed ultrasonically in an ice bath for 10 min, and the supernatant was collected by centrifugation. One unit of enzyme activity was defined as the amount of enzyme required to generate 1 μmol of product per minute.

#### 2.6.2. Determination of toluene dioxygenase activity

The determination was carried out using a UV spectrophotometer. An appropriate amount of cell crushing solution was taken at 30°C and quickly added to the solution of indole to detect the production of indigo at 600 nm.

#### 2.6.3. Determination of catechol 1,2 dioxygenase activity

The determination was carried out using a UV spectrophotometer. An appropriate amount of cell crushing solution was taken at 30°C and quickly added to the solution of catechol to detect the production of cis, cis-muconic acid at 260 nm.

#### 2.6.4. Determination of catechol 2,3 dioxygenase activity

The determination was carried out using a UV spectrophotometer. An appropriate amount of cell crushing solution was taken at 30°C and quickly added to the solution of catechol to detect the production of 2-hydroxymucofuranic acid semialdehyde at 375 nm.

## 3. Results and discussion

### 3.1. Characterization

#### 3.1.1. Zeta potential analysis

**Figure 1:**
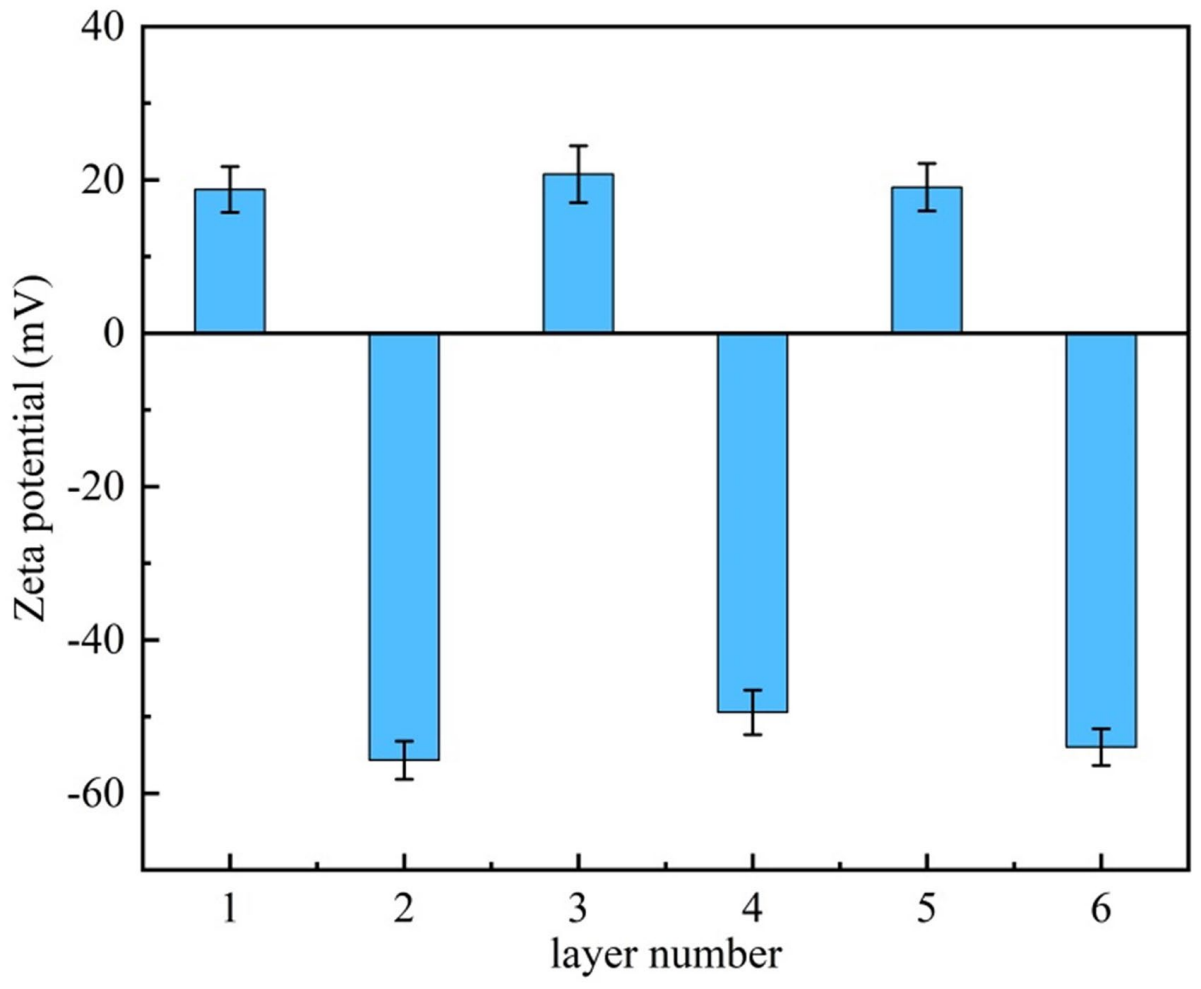
The surface Zeta-potential of SiO_2_ microparticles after the deposition of each layer.

The Zeta potential of the SiO_2_ microparticles surface after each deposition of the polyelectrolyte was measured to determine whether the CTS/PLA were deposited successfully (Dai et al., 2021). The surface potentials of SiO_2_ are shown in Figure 1. With the alternative deposition of CTS and PLA, the Zeta potential of the SiO_2_ surface showed periodic changes at 20 mV and −57 mV. The results showed that two polyelectrolytes with opposite charges were successfully deposited on the SiO_2_ surface.

#### 3.1.2. FTIR analysis

**Figure 2:**
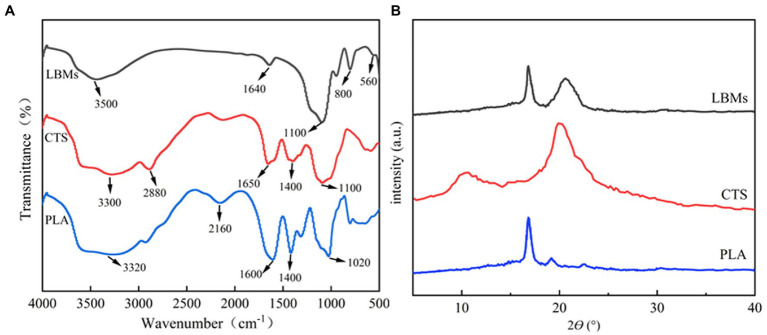
The FT-IR spectra of CTS, PLA, and LBL microcapsules **(A)** and XRD patterns of CTS, PLA, and LBL microcapsules **(B)**.

The FTIR spectra of CTS, PLA, and LBL microcapsules are shown in Figure 2A. The FTIR analysis peaks were measured in the range of 500–4,000 cm^−1^. According to the results, the characteristic peaks of CTS appeared at 3,300, 2,880, 1,650, and 1,400, corresponding to the N-H stretching vibration peak, C-H stretching vibration peak, amino, and carboxyl in the compound, respectively (Han et al., 2010; Bahraminegad et al., 2021). The characteristic peaks of PLA appeared at 1,400 and 1,020 cm^−1^, corresponding to the carboxyl group and C-O-C antisymmetric stretching vibration in the compound, respectively (Ye et al., 2018). The LBL microcapsules showed amino and C-O-C antisymmetric stretching vibrations at 1,640 and 1,100 cm^−1^, confirming the existence of CTS and PLA in the composite. The characteristic peaks intensity of amino and carboxyl groups in LBL microcapsule materials is weakened, indicating that the amino groups of CTS and the carboxyl groups of PLA in the LBL microcapsule materials underwent an amidation reaction (Han et al., 2018), further demonstrating the successful preparation of LBL microcapsules.

#### 3.1.3. XRD analysis

The XRD spectra of CTS, PLA, and LBL microcapsules are shown in Figure 2B. The CTS showed two broad peaks at around 10.2° and 20.6° related to the hydrated and anhydrous crystals, respectively (Li et al., 2009). The PLA showed two peaks at around 16.9° and 19.1° related to the (200) and (203) crystal faces (Yin, 2018). The LBL microcapsules showed two peaks at around 16.9° and 20.6°, corresponding to the characteristic peak of CTS and PLA, respectively, but the peak intensity was weakened. These results suggested that a degree of interaction between CTS and PLA occurred during the LBL microcapsules formation, altering the crystalline structure of CTS and PLA.

#### 3.1.4. SEM and TEM analysis

**Figure 3:**
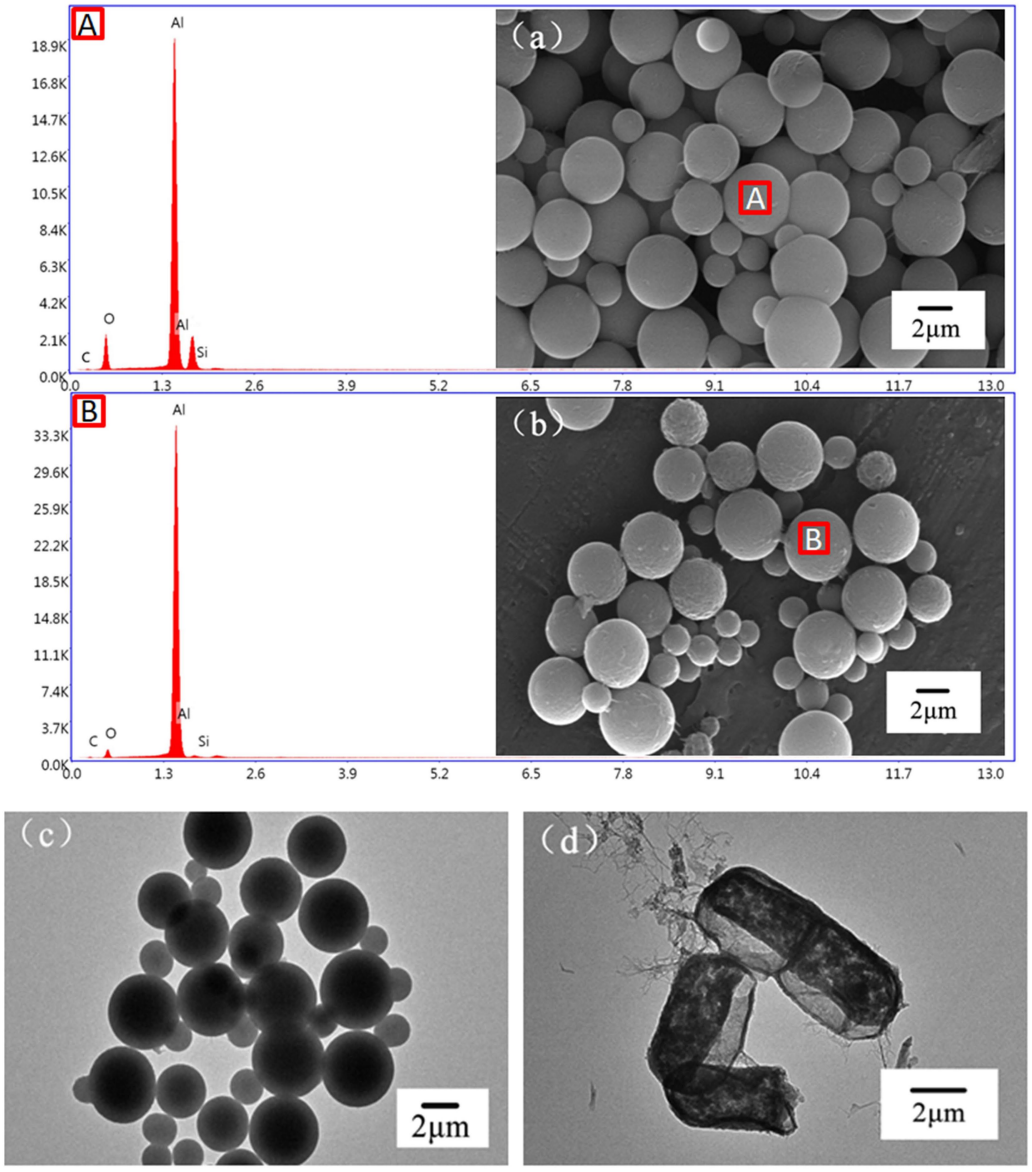
SEM and EDS images of LBMs with **(A)** or without **(B)** SiO_2_ template, TEM images of LBMs with **(C)** or without **(D)** SiO_2_ template.

The SEM images of the LBMs are shown in Figures 3A,B. It can be seen from the figure that the microcapsules with SiO_2_ templates were regular spherical structures with diameters of around 5 μm. The selected bacteria were nanoscale in size, which was much smaller than the microcapsules, indicating that the experiment was theoretically feasible. After the SiO_2_ templates were removed, the LBMs still maintained a regular spherical structure. The surface of the LBMs became rough, facilitating contact with toluene and improving the removal of toluene. The EDS images show that the Si content was high before the removal of the SiO_2_ template, while the Si content decreased dramatically to near zero after the removal of the SiO_2_ template, indicating that the template was successfully removed. The highest Al content was due to the fact that the SEM was carried out on aluminum foil.

The TEM images of the LBMs are shown in Figures 3C,D. The LBMs showed a deep black core and a slightly brighter capsule wall (Figure 3C), indicating the existence of the SiO_2_ templates. The shape of the LBMs changed slightly after the template removal with dilute HF acid due to high-speed centrifugation during collection. However, the LBMs did not collapse or break, indicating that the LBMs had good strength. The capsule wall was clearly visible and consisted of alternating deposition of CTS and PLA (Figure 3D). The deep black cores disappeared, indicating that the SiO_2_ templates were removed and replaced by bacteria. These results verified the removal of the SiO_2_ templates from the LBMs and bacteria were encapsulated successfully.

#### 3.1.5. Optical microscope analysis

**Figure 4:**
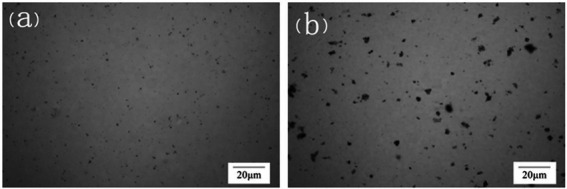
Electron microscope images of free bacteria **(A)** and LBMs **(B)**.

In order to further confirm the encapsulation of microorganisms by LBMs, the distribution of microorganisms in LBMs was observed by living bacteria stained with safranine dye solution. As shown in Figure 4, the distribution of free bacteria was relatively uniform, while the distribution of bacteria encapsulated by LBMs was more concentrated, indicating that the microorganisms were successfully encapsulated in the microcapsules.

#### 3.1.6. Bet analysis

**Figure 5:**
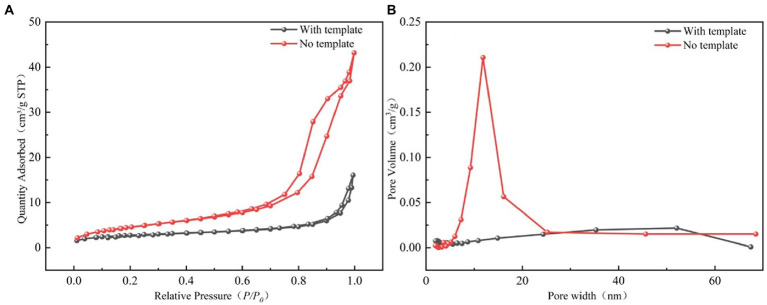
Nitrogen adsorption–desorption isotherms of the LBMs **(A)**, and mesopore size distributions of the LBMs using BJH model **(B)**.

The N_2_ adsorption–desorption isotherms and pore size distribution for the LBMs with and without SiO_2_ templates are shown in Figure 5. The specific surface area, pore volume, and other related parameters of LBMs are shown in Table 1. It can be seen from Figure 5A that the adsorption–desorption isotherms of LBMs were the class V isotherms, indicating that they had a large number of mesoporous structures (Muttakin et al., 2018). It can be seen from Figure 5B that the vast majority of the mesoporous in the LBMs were between 2 and 50 nm, which further confirmed that the LBMs contained mainly mesoporous structures. It can be seen from Table 1 that the pore specific surface area of LBMs with SiO_2_ templates accounted for 56% of the total BET surface area, and the mesoporous proportion was as high as 77%. However, the pore specific surface area of LBMs without SiO_2_ templates accounted for 84% of the total BET surface area, and the mesoporous proportion was as high as 90%. The existence of a large number of mesoporous structures was conducive to the survival of microorganisms and the delivery of pollutants and metabolites.

**Table 1:** Surface area, pore size and pore volume parameters for LBL microcapsules.

Sample	*S*_BET_ (m^2^ g^−1^)	*S*_pore_ (m^2^ g^−1^)	*S*_pore_/*S*_BET_ (%)	*V*_mes_ (cm^3^ g^−1^)	*V*_mes_ (cm^3^ g^−1^)	*V*_t_ (cm^3^ g^−1^)	*V*_mes_ /*V*_t_ (%)	Hole size (nm)
LBL microcapsules with SiO_2_ templates	9.68	5.39	56	0.0185	0.0037	0.0241	77	9.9718
LBL microcapsules without SiO_2_ templates	17.19	14.39	84	0.0576	0.0042	0.0638	90	14.8535

*S*_BET_: Measured using N_2_ adsorption with the Brunauer–Emmett–Teller (BET) method. *S*_pore_: Pore surface area. *V*_mes_, *V*_mes_ - Mesopore, micropore volume. *V*_t_: Total pore volume. Hole size: Pore size in diameter means average values.

After the SiO_2_ templates were removed, the specific surface area of LBMs nearly doubled to 17.19 m^2^/g, demonstrating that the SiO_2_ templates were successfully removed. The average pore size of LBMs increased from 9.9718 to 14.8535 nm when the SiO_2_ templates were removed, indicating that the pore structures increased. The hollow structure created by removing the SiO_2_ templates could provide a microenvironment that is conducive to the survival and multiplication of bacteria.

### 3.2. Environmental adaptation of LBL microcapsules immobilized EGI312

#### 3.2.1. The effects of the initial toluene concentration on biodegradation

**Figure 6:**
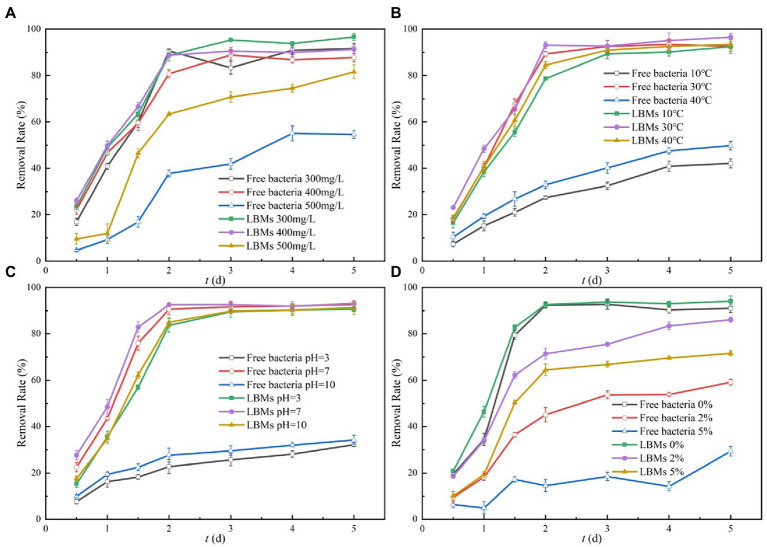
Effect of initial toluene concentrations **(A)**, temperature **(B)**, pH **(C)**, and salinity **(D)** on the toluene removal rate with times.

The effects of initial toluene concentration on toluene degradation by free EGI312 and LBMs were studied. The changes of toluene removal rate with initial concentration are shown in Figure 6A. In the selected initial concentration range of toluene, the removal rate of toluene by the LBMs system was higher than that of the free bacteria system. When the initial concentrations of toluene were 300 and 400 mg/L, the removal rates of toluene in the two systems were similar. This may be due to the fact that 300 mg/L of toluene essentially had no stressful effect on the growth and reproduction of EGI312. However, when the initial concentration of toluene was 500 mg/L, the removal rate of toluene by the LBMs system was significantly higher than that by the free bacteria system. The removal rate of toluene by the LBMs system was up to 83% on the 5th day, while the removal rate of the free bacteria system was only 52%.

The above results showed that a high initial concentration of toluene reduced the removal rate of toluene by bacteria, which may be due to the biological toxicity of high concentrations of toluene and its intermediate metabolites. These results were likely due to the high concentration of toluene damaging the microbial cells, which causes the selective permeability of the cytomembrane and enzyme inactivation. In addition, the toxicity of toluene at higher concentrations could lead to metabolism inhibition during toluene degradation, resulting in lower removal efficiency (Hsieh et al., 2008; Chen et al., 2021). For the LBMs system, the extracellular polymeric substances (EPS) from EGI312 could be combined with CTS or PLA to generate a stable ground material that effectively slows down the increasing rate of bacterial cell membrane permeability, which could protect the bacteria. This will also enhance the toluene degradation capability at high toluene concentrations for the LBMs system.

#### 3.2.2. The effects of temperature on biodegradation

Generally, temperature fluctuation in the actual environment can affect the degradation of microorganisms. The effects of temperature on the free bacteria system and the LBMs system were studied. The experimental results are shown in Figure 6B. The bacterial degradation of toluene is optimal at a temperature of 30°C. There was no significant difference in the removal of toluene between the free bacteria system and the LBMs system. However, the activity of bacteria was negatively affected at a temperature of 10°C or 40°C and hence hindered its biodegradation capability. The removal rate of toluene by the LBMs system was much higher than that of the free bacteria system. When the temperature decreased from 30 to 10°C, the toluene degradation rate with the free bacteria system decreased from 91 to 39% on the 5th day, while the LBMs system only decreased from 95 to 89%. Similarly, at 40°C, the toluene degradation rate with the free bacteria system decreased from 91 to 44% on the 5th day, while the LBMs system only decreased from 95 to 90%. Generally speaking, the removal rate of toluene by the LBMs system was more than twice that of free bacteria at 10 and 40°C.

From the above results, it can be seen that the LBMs system had better tolerance to high temperature environments or low temperature environments than free bacteria, thus improving the degradation rate of toluene by bacteria. Temperature affects the removal rate of toluene by bacteria in two ways. Firstly, temperature was closely related to the growth rate of bacteria and the activity of toluene-degrading enzymes. The growth and reproduction of bacteria were greatly inhibited at unfavorable temperatures, as was the activity of the extracellular enzymes produced by the bacteria. And an important biodegradation mechanism of toluene by EGI312 in this study may be the action of extracellular enzymes. Secondly, temperature affected the mass transfer rate and bioavailability of toluene (Su et al., 2001; Liu, 2014). At high temperatures, the membrane toxicity of toluene has increased, which usually results in the rupture of the bacterial cytosol (Ren and Huang, 2001). Another explanation is that the increase in the temperature caused a decline in oxygen solubility, which was not conducive to the metabolic activity of aerobic microorganisms (Liu et al., 2012). On the other hand, exposure to lower temperatures is known to slow down bacterial activity. When the bacteria are immobilized by LBL microcapsules, they separate the microorganisms from the surrounding environment to form a mild microenvironment. Therefore, the LBMs system showed enormously better thermal stability than the free bacteria system.

#### 3.2.3. The effects of pH on biodegradation

Environmental pH is an important factor affecting the growth of bacteria. The effects of pH on the removal rate of toluene by the free bacteria system and the LBMs system were investigated by comparative experiments, and the results are shown in Figure 6C. At pH = 7, the toluene removal rates of the free bacteria system and the LBMs system were basically the same, reaching more than 90%. When the pH decreased from 7 to 3, the toluene removal rate of the free bacteria system decreased from 93 to 35%. In contrast, the toluene removal rate of the LBMs system only decreased from 93 to 90%. The toluene removal rate of the LBMs system was 2.5 times that of the free bacteria system. Similarly, at pH = 10, the toluene removal rate of the LBMs system was 90%, while that of the free bacteria system was only 36%.

The above results showed that the free bacteria system was greatly inhibited in an acidic or alkaline environment, which greatly reduced the removal rate of toluene by bacteria. This may be due to the fact that extreme pH could denature bacterial biomolecules such as proteins or nucleic acids, thus affecting their activity (Zhang et al., 2021). On the other hand, pH could cause changes in the cell membrane potential. Cell membrane potential was closely related to membrane permeability, which in turn affected the uptake and utilization of substrates by bacteria (Al-Hawash et al., 2018). The LBMs system was not sensitive to the changes in external pH and could maintain a high removal rate over a relatively wide pH range. This is because CTS and PLA hydrolyze very slowly under acidic conditions, while PLA hydrolyzes faster but chitosan does not dissolve under alkaline conditions (Chen et al., 2020; Ren et al., 2022), thus ensuring the stable operation of LBMs. Meanwhile, the rate of hydrolysis of PLA is closely related to temperature and accelerates with increasing temperature (Ren et al., 2022). Complete hydrolysis is possible in the range of 173–200°C, while at lower temperatures, the hydrolysis rate is rather slow (Cristina et al., 2018). Groundwater temperatures are apparently not that high (Cavelan et al., 2022). This also ensures the smooth operation of the LBM.

#### 3.2.4. The effects of salinity on biodegradation

The effect of salinity on the bacteria is shown in Figure 6D, where the effect of salinity in the range of 0 to 5% on the free bacteria system and the LBMs system was evaluated. The results showed that the 5 days toluene removal rate of the free bacteria system decreased from 90 to 59% as the salinity increased from 0 to 2%, and with the salinity increased to 5%, the removal rate of toluene was further decreased to 26%. In contrast, the 5d degradation of toluene by the LBMs system was initially reduced from 93 to 85% and then to 70%. Salinity affects its activity by influencing the osmotic pressure of cells. High salinity in the environment disrupts the cell membrane and the enzyme system in the microorganism (Wu et al., 2011), which in turn affects the removal rate of toluene by the bacteria. The results of this study showed that the LBMs system had greater adaptability to salt stress because it can provide a certain barrier effect for the microorganisms, thus avoiding the stressful effect on the bacteria due to elevated osmotic pressure.

Although the removal effects of the free bacteria system and the LBMs system under the optimal external condition (T = 30°C, pH = 7, Salinity = 0%) in this experiment did not differ significantly. However, in the real environment, free bacteria are easily washed away by water, which does not facilitate their settlement and weakens the microbial treatment effect (Boufadel et al., 2016). However, LBMs can agglomerate microorganisms into clusters and help them to settle better for a better microbial treatment effect.

### 3.3. Microbial degradation kinetics

**Figure 7:**
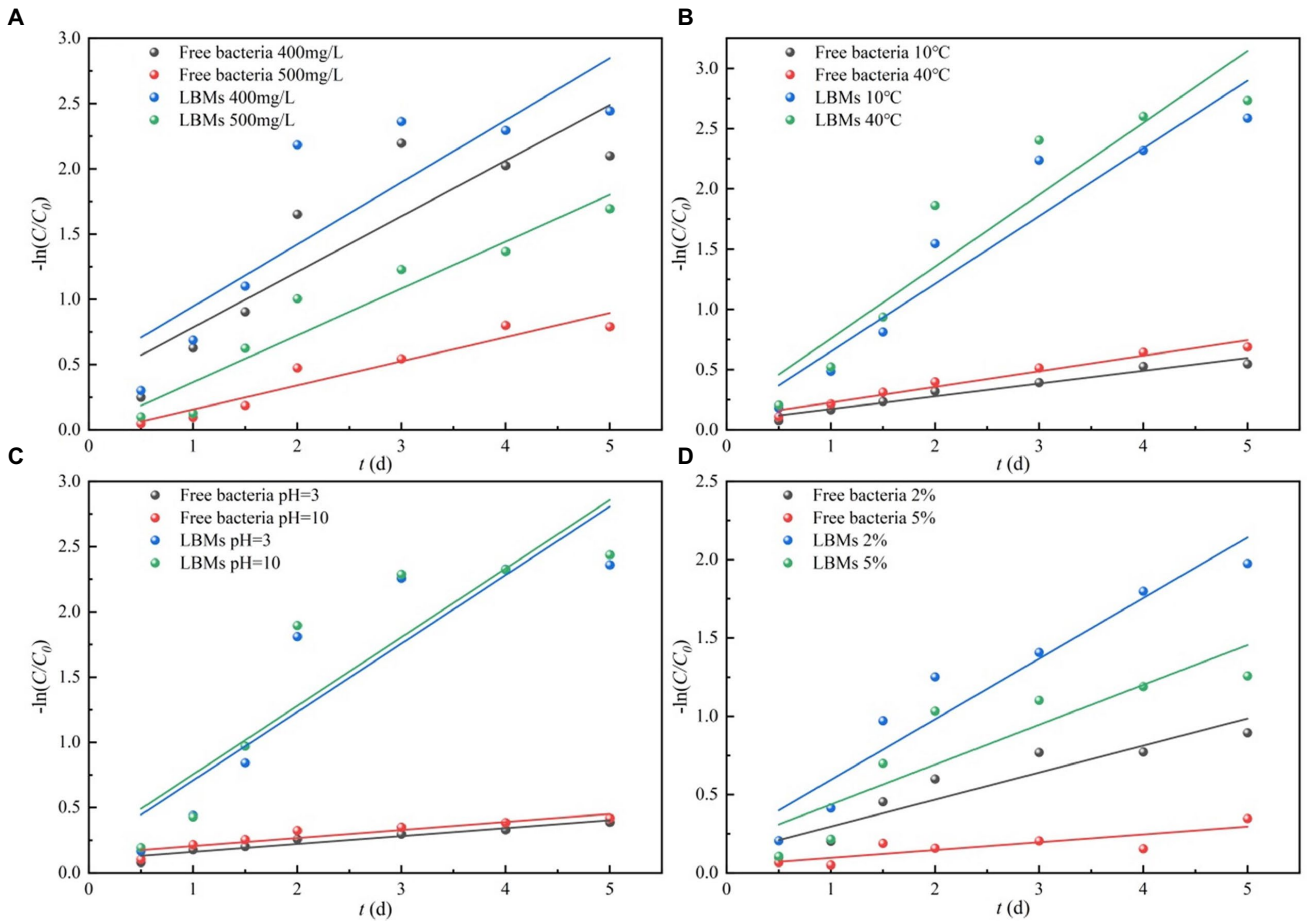
First-order kinetic fitting of toluene degradation by free bacteria and LBMs under harsh environmental conditions initial toluene concentrations **(A)**, temperature **(B)**, pH **(C)** and salinity **(D)**.

The first-order kinetic model was adopted to perform linear fitting of experimental data. The fitting results are shown in Figure 7 and Table S2.

(1) $$\mathrm{The first-order kinetic model}C=C_{0}e^{-kt}$$

(2) $$\mathrm{The half}-\mathrm{life was calculated}\;\mathrm{as}\;t_{1/2}=\frac{1}{k}\ln2$$

where *C*_0_ is the initial toluene concentration; *C* is the toluene concentration at time *t*; *t* is the biodegradation time; *t*_1/2_ is the half-life of toluene biodegradation; *k* is the degradation rate constant.

Figure 7 shows the microbial degradation kinetics of toluene solution under different environmental conditions, and the relevant fitting parameters are given in Table 2. In harsh environmental conditions, the degradation rate constants of toluene by the LBMs system were greater than those of the free bacteria systems, and the half-life of the LBMs system was significantly less than those of the free bacteria system. These results verified that the advantages of the LBMs for toluene biodegradation were therefore prominent. This was likely because the LBL microcapsules can provide bacteria protection from the pollutant.

**Table 2:** Kinetic parameters of toluene removal by free bacteria and LBMs.

Free bacteria				LBMs			
Sample	*k*/d^−1^	*t*_1/2_/d	*R* ^2^	Sample	*k*/d^−1^	*t*_1/2_/d	*R* ^2^
400 mg/L	0.4260	1.6271	0.8065	400 mg/L	0.4757	1.4571	0.7826
500 mg/L	0.1846	3.7549	0.9245	500 mg/L	0.3590	1.9308	0.9176
10°C	0.1060	6.5391	0.9605	10°C	0.5624	1.2325	0.9111
40°C	0.1295	5.3525	0.9648	40°C	0.7696	0.9007	0.8826
pH = 3	0.0606	11.4381	0.9241	pH = 3	0.5247	1.3210	0.8242
pH = 10	0.0616	11.2524	0.8581	pH = 10	0.5260	1.3178	0.8202
2%	0.1726	4.0159	0.8736	2%	0.3874	1.7892	0.9211
5%	0.0499	13.8907	0.9211	5%	0.2546	2.7225	0.8912

### 3.4. The change in bacteria survival and death

**Figure 8:**
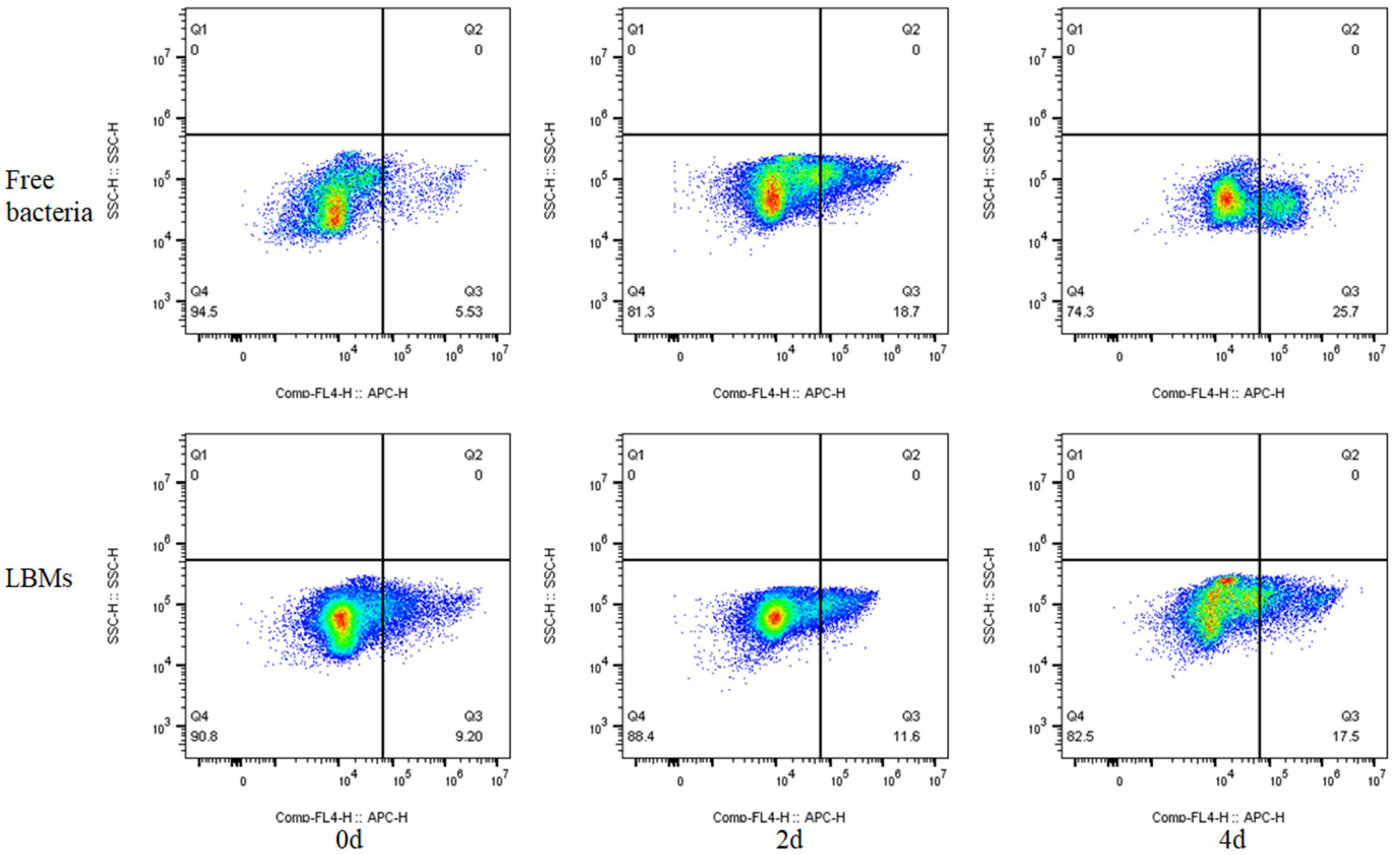
Bacteria viability quantified using flow cytometry in free bacteria and LBMs systems (The initial toluene concentration was 500 mg L^−1^).

The activity of free bacteria and LBMs systems was detected during the 500 mg/L toluene degradation process. The results are shown in Figure 8, where the Q3 region represents the percentage of dead bacteria and the Q4 region represents the percentage of surviving bacteria. The changes of the proportion of the two regions of bacteria can directly reflect the inhibitory action of toluene on EGI312 bacteria. As can be seen from the Figure 8, the proportion of active bacteria gradually decreased with the extension of the degradation time. The percentage of live bacteria in the free bacteria system decreased rapidly from initial 94.5–81.3% on the 2nd day and further to 74.3% on the 4th day. However, the percentage of live bacteria in the LBMs system only decreased from initial 90.8–88.4% on the 2nd day, and remained 82.5% on the 4th day. The results further indicated that the effect of toluene at the high initial toluene concentration (500 mg/L) on the cell membrane of free bacteria is obvious, leading to membrane structure damage, which causes the bacteria death and the degradation of toluene more slowly. However, the LBMs system can weaken this damage effect and reduce the rate of bacteria death, causing the bacteria to degrade toluene at a faster rate.

### 3.5. Effect of environmental factors on the activity of enzyme

The activities of several toluene-degrading enzymes were measured in the free bacteria system and the LBMs system under different environmental conditions. They were measured to speculate on the protective mechanism of LBL microcapsules against bacteria and the biodegradation pathway of toluene. The experimental results are shown in Supplementary Figures S1–S4. Toluene dioxygenase, catechol 1,2-dioxygenase, and catechol 2,3-dioxygenase, the key enzymes for biodegradation of toluene (Woo et al., 2000), were detected in all two systems. The difference in the enzymatic activities between the two systems was small under mild external conditions (initial toluene concentration of 300 mg/L, pH = 7, temperature = 30°C, salinity = 0%). However, when the bacteria were stressed by unfavorable external environmental conditions, the activities of the three enzymes of the LBMs system were significantly higher than the free bacteria system.

The activities of several enzymes on the 5th day are shown in Table 3. Regardless of the external conditions, the activity of toluene dioxygenase in the LBMs system reached 0.7 mg indigo min^−1^, while the free bacteria system was only about 0.5 mg indigo min^−1^. The activities of catechol 1,2-dioxygenase and catechol 2,3-dioxygenase were more influenced by environmental conditions, and the activities of these two enzymes in the LBMs system could reach 2–4 times that of the free bacteria system. Overall, the activities of several enzymes in the degradation of toluene by the LBMs system were much higher than the free bacteria system, which may be related to the fact that the LBL microcapsules formed sufficient protection for the microorganisms. The free bacteria were in direct contact with the external environment, and the unfavorable external environment (high pollutant concentration, pH, temperature, salinity) had a direct coercive effect on the bacteria, which in turn had a negative impact on the enzymatic activity of the bacteria. On the one hand, high toluene concentrations caused selective penetration of toluene through the cell membrane into the cell, which in turn destroyed the intracellular enzymes and cell integrity. On the other hand, the efficient catalytic degradation of all enzymes is based on the appropriate pH and temperature. Too high or too low pH and temperature are not conducive to the action of microbial enzymes and may even destroy their structures to deactivate them. The LBL microcapsules separate the external environment from the bacteria, creating a separate microenvironment. This reduces the effect of the external environment on the microbial enzyme activity and greatly improves the adaptability of the microorganisms to the stressful external environment.

**Table 3:** Activity of TDO, C1,2D, and C2,3D in the free bacteria system and the LBMs system at the 5th day under different conditions.

Free bacteria				LBMs			
Sample	TDO activity/U	C1,2D activity/U	C2,3D activity/U	Sample	TDO activity/U	C1,2D activity/U	C2,3D activity/U
300 mg/L	0.769	0.029	0.017	300 mg/L	0.808	0.051	0.041
400 mg/L	0.51	0.016	0.011	400 mg/L	0.769	0.044	0.037
500 mg/L	0.348	0.013	0.009	500 mg/L	0.784	0.049	0.04
10°C	0.687	0.021	0.047	10°C	0.846	0.074	0.079
30°C	0.729	0.054	0.068	30°C	1.009	0.075	0.089
40°C	0.669	0.03	0.048	40°C	0.957	0.079	0.081
pH = 3	0.708	0.009	0.088	pH = 3	0.733	0.022	0.212
pH = 7	0.836	0.018	0.169	pH = 7	0.942	0.031	0.336
pH = 10	0.701	0.007	0.078	pH = 10	0.731	0.027	0.231
0%	0.783	0.034	0.021	0%	0.811	0.045	0.038
2%	0.654	0.011	0.016	2%	0.807	0.044	0.034
5%	0.632	0.012	0.007	5%	0.797	0.032	0.029

### 3.6. Analysis of biodegradation pathway of toluene

**Figure 9:**
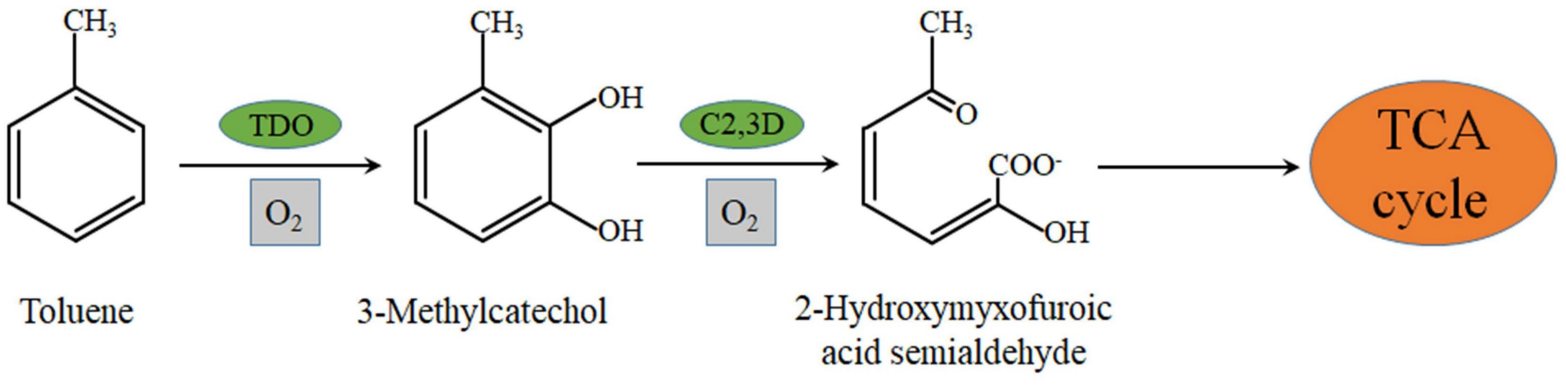
Toluene degradation pathway of EGI312 derived from analysis of key enzymes.

In a previous study, we examined the activities of toluene dioxygenase, catechol 1,2 dioxygenase, and catechol 2,3 dioxygenase in the crude enzyme solution of strain EGI312 and all three enzymes were detected. The enzymatic activity of catechol 2,3-dioxygenase was much higher than that of catechol 1,2 dioxygenase. Thus, we speculate that catechol 2,3-dioxygenase is mainly responsible for the degradation process of toluene, and the degradation pathway of toluene by strain EGI312 was inferred according to the available research results. As shown in Figure 9, toluene is first converted to catechol by the action of dioxygenase, and then the benzene ring is opened by the catalytic action of catechol 2,3-dioxygenase. The long-chain organic compounds obtained after opening the benzene ring are further decomposed into small molecules, such as pyruvic acid and acetaldehyde, by the actions of various dehydrogenases and hydrolases (Parales et al., 2008). Finally, the resulting small molecular compounds are oxidized *via* the Krebs cycle (Deng et al., 2017; Feng et al., 2021).

## 4. Conclusion

The LBMs were successfully prepared by depositing CTS and PLA layers onto the surface of SiO_2_ and were used for the first time for toluene abatement. The environmental-friendly CTS and PLA offer the possibility of practical applications for LBMs. The biodegradation experiments under unfavorable environmental factors showed that the LBMs was highly tolerant to the unfavorable external environment and could maintain a high degradation rate under high pollutant concentration, high/low temperature, high/low pH, or high salinity conditions. This is an essential feature for the possible industrial use of biotechnology for the remediation of toluene contamination. Flow cytometry analysis showed that LBL microcapsules could effectively reduce the death rate of the bacteria, which could protect the degrading bacteria. The enzyme activity assay revealed that LBMs can effectively shield microbial enzymes from negative effects caused by external harsh environmental conditions. The results of this study provide a practical basis and theoretical rationale for the practical application of biotechnology, further demonstrating that encapsulating bacteria with LBL microcapsules is an effective bioremediation strategy for tackling toluene contamination in groundwater.

## Data availability statement

The original contributions presented in the study are included in the article/Supplementary material, further inquiries can be directed to the corresponding authors.

## Author contributions

HL: data curation, formal analysis, investigation, methodology, validation, writing–original draft, writing–review and editing. YY: investigation, software, and validation. YL: software and validation. XF: investigation, software, and visualization. QL: investigation, supervision, and visualization. XN: investigation, validation, and visualization. YM: conceptualization, methodology, project administration, resources, and writing–review and editing. AL: conceptualization, supervision, and writing–review and editing. All authors contributed to the article and approved the submitted version.

## Funding

This work was supported by the Shandong Provincial Natural Science Foundation (ZR2020MD108 and ZR2020ZD19) and the Program of Zibo School City Fusion (2021JSCG0012).

## Conflict of interest

Author YL was employed by Shandong Academy of Environmental Science Co., Ltd.

The remaining authors declare that the research was conducted in the absence of any commercial or financial relationships that could be construed as a potential conflict of interest.

## Publisher’s note

All claims expressed in this article are solely those of the authors and do not necessarily represent those of their affiliated organizations, or those of the publisher, the editors and the reviewers. Any product that may be evaluated in this article, or claim that may be made by its manufacturer, is not guaranteed or endorsed by the publisher.

## Supplementary material

The Supplementary material for this article can be found online at: https://www.frontiersin.org/articles/10.3389/fmicb.2023.1122966/full#supplementary-material
