# Supplementary material for: Bioenhanced degradation of toluene by layer-by-layer self-assembled silica-based bio-microcapsules
Source: Front Microbiol. 2023 Feb 20;14:1122966. doi: 10.3389/fmicb.2023.1122966 (PMC9986300; doi:10.3389/fmicb.2023.1122966)
Supplement: Supplementary file 1 [file Table_1.docx]

Bioenhanced degradation of toluene by layer-by-layer self-assembled silica-based bio-microcapsules

**Hongyang Lin^a^, Yang Yang^a^, Yongxia Li^b^, Xuedong Feng^c^, Qiuhong Li^d^, Xiaoyin Niu^c^, Yanfei Ma^c,*^, Aijv Liu^c*^**

^a^ *School of Agricultural Engineering and Food Science, Shandong University of Technology, Zibo 255049, China*

^b^ *Shandong Academy of Environmental Science Co.,Ltd, Jinan 250000, China*

^c^ *School of Resources and Environmental Engineering, Shandong University of Technology, Zibo 255049,China*

^d^ *School of Materials Science and Engineering, Shandong University of Technology, Zibo 255049, China*

**Corresponding author.*

*E-mail address: mayanfei@sdut.edu.cn (Y. F. Ma)；aijvliu@sdut.edu.cn （A. J. Liu）*


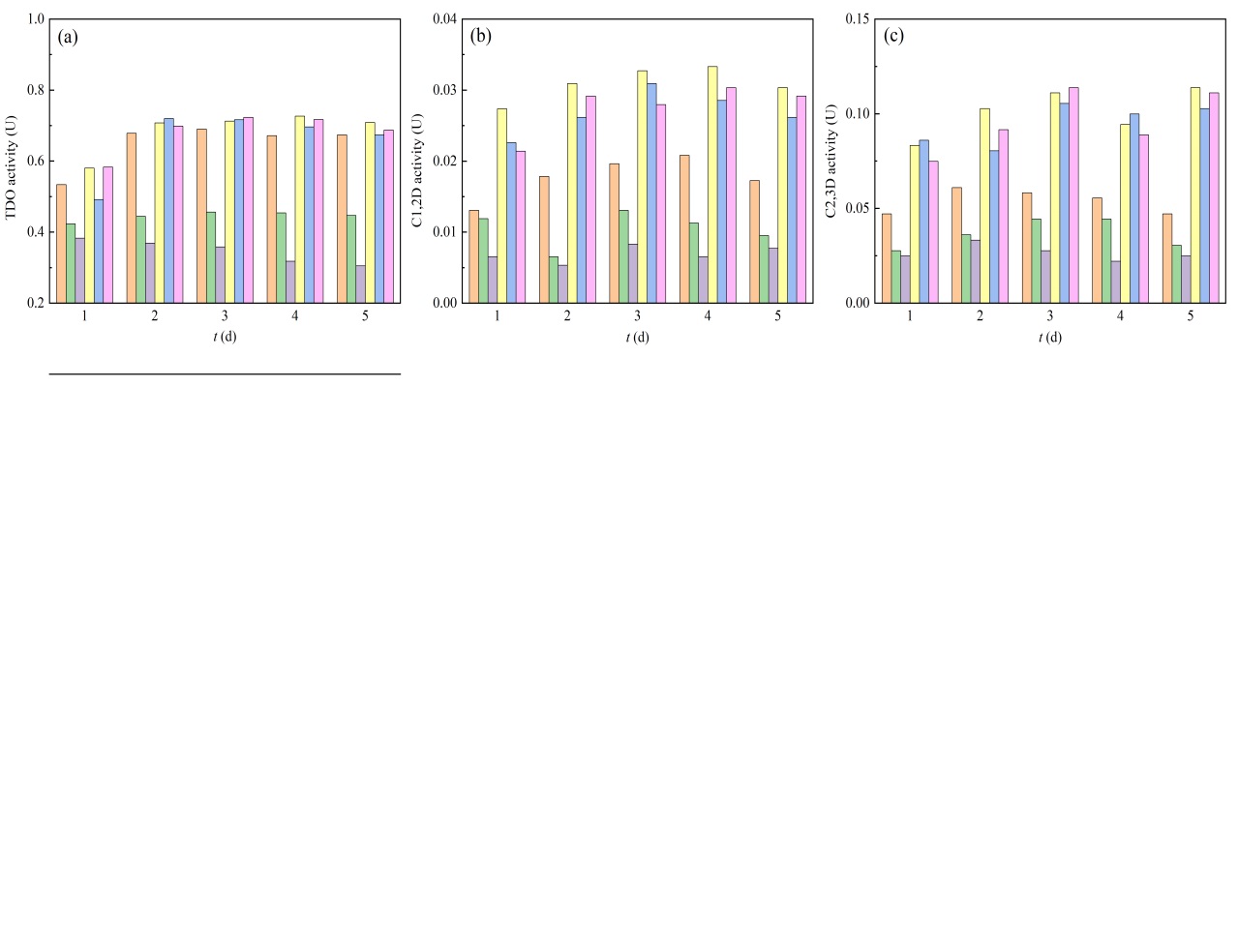


Figure S1. Activity of (a) toluene dioxygenase, (b) catechol 1,2 dioxygenase, and (c) catechol 2,3 dioxygenase in the free bacteria system and the LBMs system at different initial toluene concentrations. ( ) Free bacteria 300mg/L; ( ) Free bacteria 400mg/L; ( ) Free bacteria 500mg/L; ( ) LBMs 300mg/L; ( ) LBMs 400mg/L; ( ) LBMs 500mg/L.
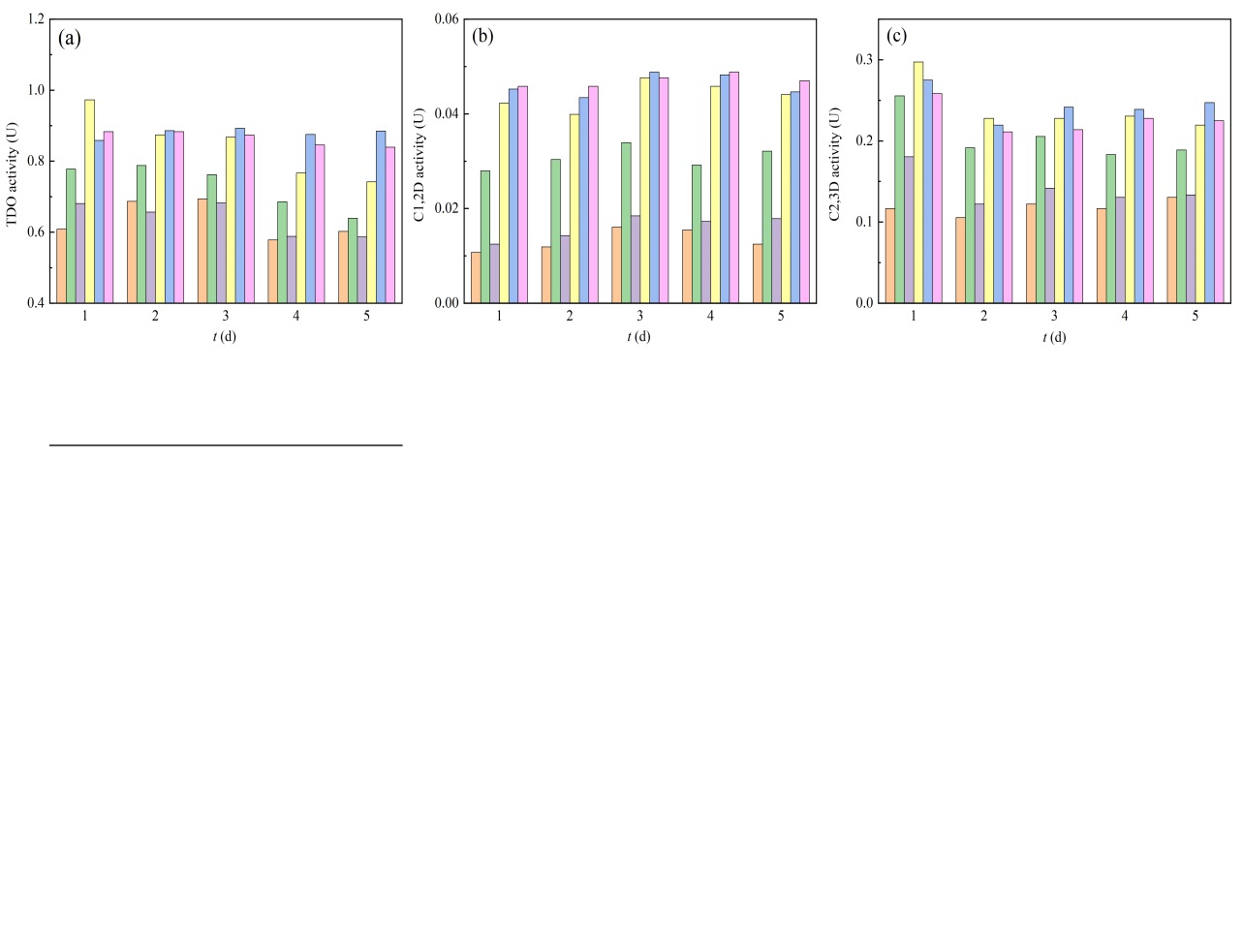


++++++++++++++++++++

Figure S2. Activity of (a) toluene dioxygenase, (b) catechol 1,2 dioxygenase, and (c) catechol 2,3 dioxygenase in the free bacteria system and the LBMs system at different initial toluene concentrations. ( ) Free bacteria 10℃; ( ) Free bacteria 30℃; ( ) Free bacteria 40℃; ( ) LBMs 10℃; ( ) LBMs 30℃; ( ) LBMs 40℃.

++++++++++++++++++++


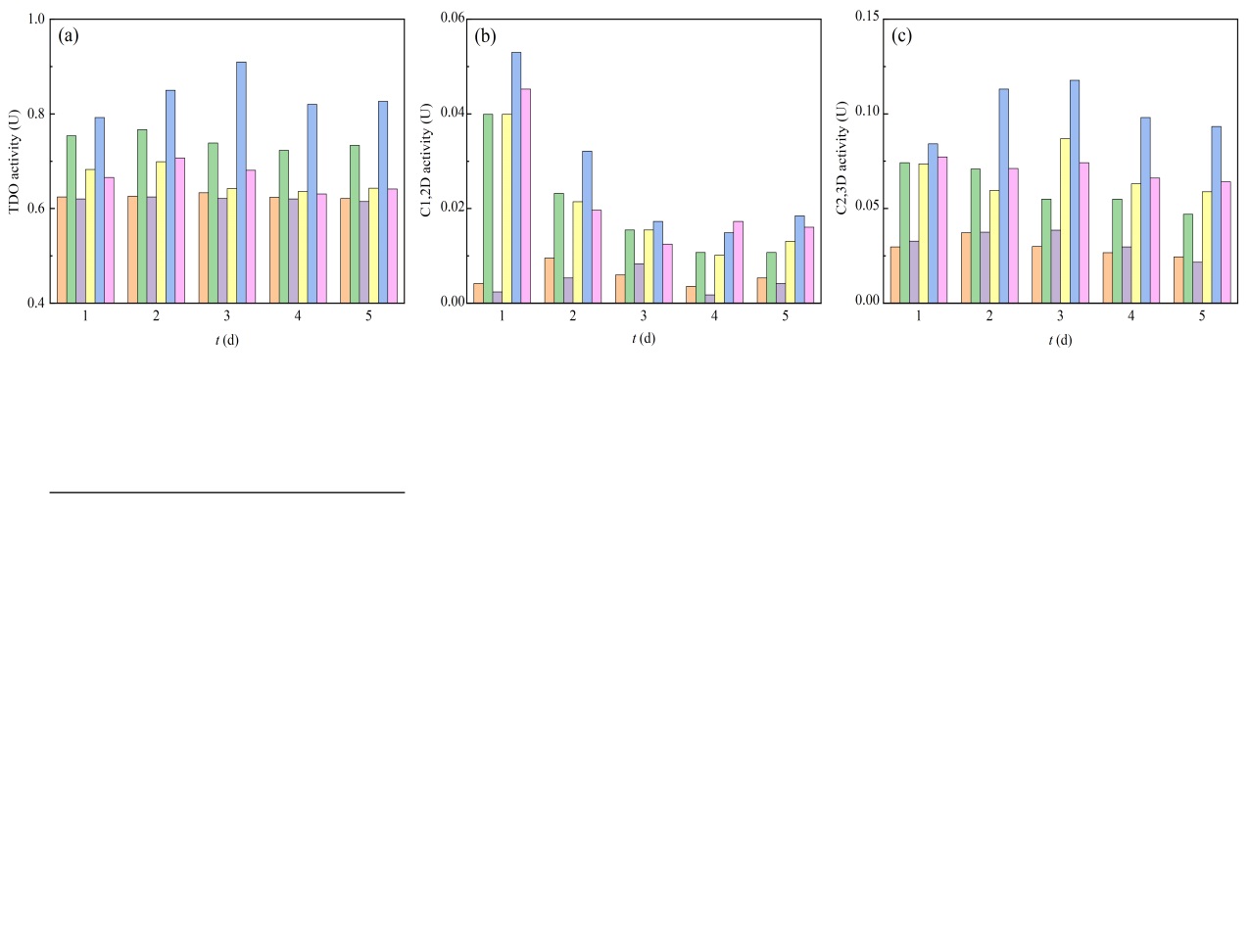


Figure S3. Activity of (a) toluene dioxygenase, (b) catechol 1,2 dioxygenase, and (c) catechol 2,3 dioxygenase in the free bacteria system and the LBMs system at different initial toluene concentrations. ( ) Free bacteria pH=3; ( ) Free bacteria pH=7; ( ) Free bacteria pH=10; ( ) LBMs pH=3; ( ) LBMs pH=7; ( ) LBMs pH=10.

++++++++++++++++++++


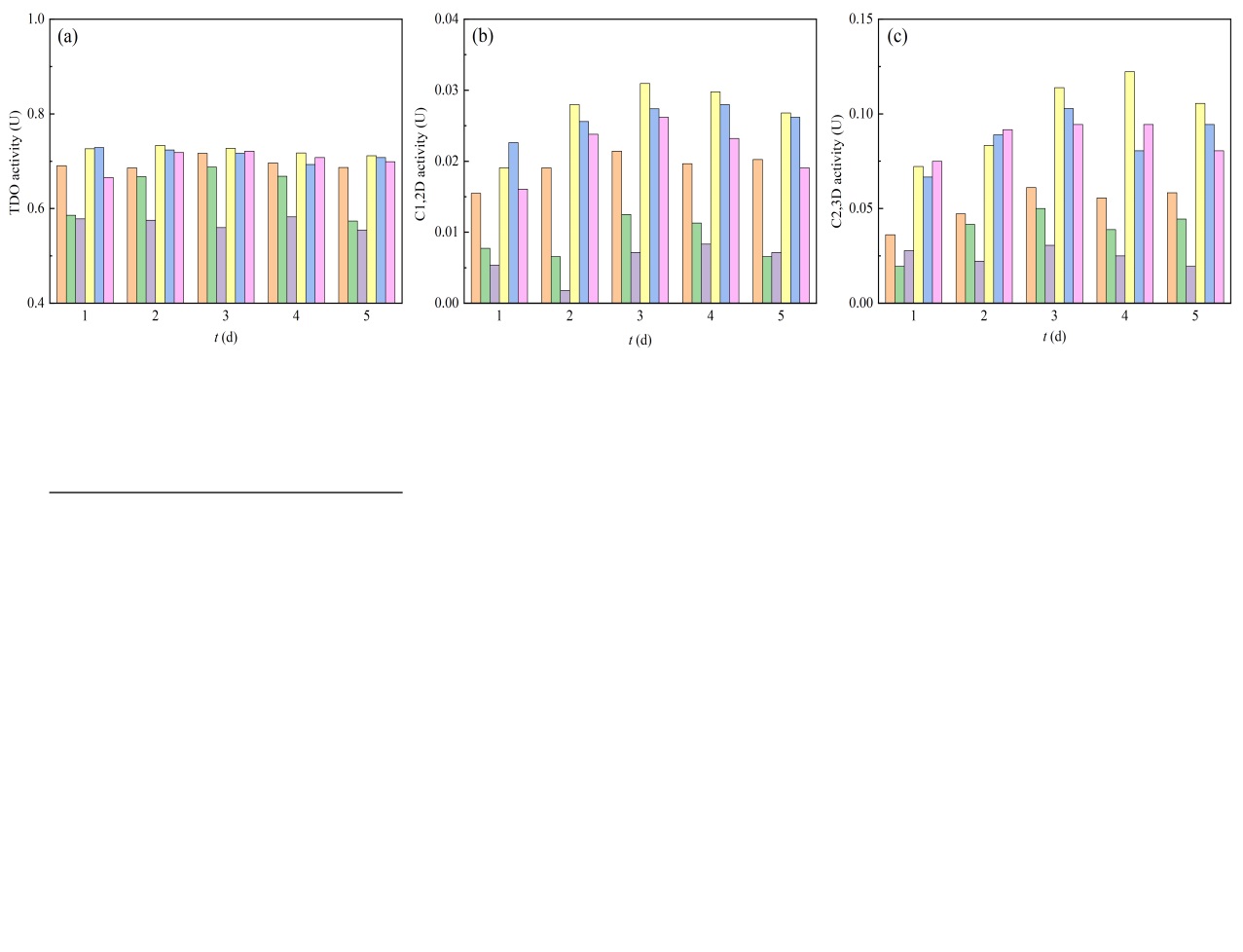


Figure S4. Activity of (a) toluene dioxygenase, (b) catechol 1,2 dioxygenase, and (c) catechol 2,3 dioxygenase in the free bacteria system and the LBMs system at different initial toluene concentrations. ( ) Free bacteria 0%; ( ) Free bacteria 2%; ( ) Free bacteria 5%; ( ) LBMs 0%; ( ) LBMs 2%; ( ) LBMs 5%.

++++++++++++++++++++
